# A Glutamic Acid-Rich Protein Identified in *Verticillium dahliae* from an Insertional Mutagenesis Affects Microsclerotial Formation and Pathogenicity

**DOI:** 10.1371/journal.pone.0015319

**Published:** 2010-12-07

**Authors:** Feng Gao, Bang-Jun Zhou, Guo-Ying Li, Pei-Song Jia, Hui Li, Yun-Long Zhao, Pan Zhao, Gui-Xian Xia, Hui-Shan Guo

**Affiliations:** 1 State Key Laboratory of Plant Genomics and National Center for Plant Gene Research (Beijing), Institute of Microbiology, Chinese Academy of Sciences, Beijing, China; 2 The Key Laboratory of Prevention and Control for Oasis Crop Disease, Shihezi University, Shihezi, Xinjiang, China; 3 Graduate University of Chinese Academy of Sciences, Beijing, China; University of Missouri-Kansas City, United States of America

## Abstract

*Verticillium dahliae* Kleb. is a phytopathogenic fungus that causes wilt disease in a wide range of crops, including cotton. The life cycle of *V. dahliae* includes three vegetative phases: parasitic, saprophytic and dormant. The dormant microsclerotia are the primary infectious propagules, which germinate when they are stimulated by root exudates. In this study, we report the first application of *Agrobacterium tumefaciens*-mediated transformation (ATMT) for construction of insertional mutants from a virulent defoliating isolate of *V. dahliae* (V592). Changes in morphology, especially a lack of melanized microsclerotia or pigmentation traits, were observed in mutants. Together with the established laboratory unimpaired root dip-inoculation approach, we found insertional mutants to be affected in their pathogenicities in cotton. One of the genes tagged in a pathogenicity mutant encoded a glutamic acid-rich protein (*VdGARP1*), which shared no significant similarity to any known annotated gene. The *vdgarp1* mutant showed vigorous mycelium growth with a significant delay in melanized microsclerotial formation. The expression of *VdGARP1* in the wild type V529 was organ-specific and differentially regulated by different stress agencies and conditions, in addition to being stimulated by cotton root extract in liquid culture medium. Under extreme infertile nutrient conditions, *VdGARP1* was not necessary for melanized microsclerotial formation. Taken together, our data suggest that *VdGARP1* plays an important role in sensing infertile nutrient conditions in infected cells to promote a transfer from saprophytic to dormant microsclerotia for long-term survival. Overall, our findings indicate that insertional mutagenesis by ATMT is a valuable tool for the genome-wide analysis of gene function and identification of pathogenicity genes in this important cotton pathogen.

## Introduction

Cotton wilt disease, caused by the phytopathogenic fungus *Verticillium dahliae* Kleb., is one of the most widespread, damaging diseases in most cotton-growing countries, including China [1], the Americas [2], [3] and Mediterranean regions [4]. Cotton wilt disease is a major threat to cotton production [5]. Colonization of cotton roots by *V. dahliae* in soil naturally leads to colonization of vascular tissues in cotton [6], [7]. Fungal hyphae grow from the root surface toward the cortical tissue that is adjacent to the stele [7] and subsequently attack the aerial parts of the plant. Vascular discoloration is a key diagnostic symptom of *V. dahliae* infection. *V. dahliae*-infected cotton plants also show symptoms including leaf vein browning and chlorosis, wilting, premature defoliation, and most severely, plant death [1], [8].

Of the two major plant-pathogenic *Verticillium* species, *V. dahliae* and *V. albo-atrum*., *V. dahliae* is especially difficult to control because it persists in soil as resting structures, called microsclerotia, for several years in the absence of a host plant [9]. *V. dahliae* is the agent of verticillium wilt diseases of hundreds of herbaceous and woody crops [10], [11]. The dormant microsclerotia are the primary infectious propagules; they germinate when they are stimulated by root exudates [9]. Recent studies in determination of infection and colonization of lettuce roots by a GFP-expressing lettuce isolate of *V. dahliae* showed that germ tube emergence from the infected root surface following inoculation extended longitudinally along root epidermal cells, and distinct appressoria formed within cell junctions and directly penetrated adjoining cells [10], [12]. As colonization progressed, elaborate hyphal networks were produced in cortical and vascular tissues and led to the eventual collapse of the infected root tip, and the mycelia advanced systemically through xylem vessels towards the taproot [12]. Phytotoxins produced by *V. dahliae* cause vascular discoloration and wilt symptoms associated with disease development [13]–[17].

Recently, screening an expressed sequence tag (EST) library from a cultured mycelium of a cotton isolate strain of *V. dahliae* revealed a cDNA that encodes a necrosis- and ethylene-inducing protein (VdNEP). This protein has been shown to play an important role in promoting vascular wilt symptoms specific to cotton leaves, by dipping cotton leaves into *E. coli*-expressed VdNEP protein solution [8]. Genes of potential importance in pathogenic growth and microsclerotial development have also been investigated via two EST libraries from cultures of a tomato isolate strain of *V. dahliae*, grown either in simulated xylem fluid medium (SXM) or under conditions that induce near-synchronous development of microsclerotia [18]. Dobinson and colleagues [19] subsequently testified that *Agrobacterium tumefaciens*-mediated transformation (ATMT) [20] could be applied in targeted gene disruption in *V. dahliae* tomato isolate. Thereafter, using ATMT and the EST databases [18], several genes involved in microsclerotial development of *V. dahliae* tomato isolate have been identified and disrupted. *VMK1*, a mitogen-activated protein kinase gene, was identified to have a role in formation of microsclerotia in *V. dahliae* [21]. Plants inoculated with *vmk1* mutants did not show vascular discoloration, suggesting that *VMK1* is essential for pathogenicity [21]. *VDH1*, a hydrophobin gene, was also confirmed to be involved in microsclerotial development but not required for pathogenicity [22], [23].

In addition to targeted gene disruption by homologous recombination, ATMT has been successfully exploited for large-scale forward genetic screens to create insertional mutants in several pathogenic species, including *Magnaporthe grisea*, *Cryptococcus neoformans*, *Colletotrichum lagenarium*, *C. higginsianum* and *Leptosphaeria maculans* [24]–[32]. Pathogenicity mutants and pathogenicity genes from *C. higginsianum*, *C. acutatum*, *M. grisea* and *L. maculans* have been successfully identified via ATMT [26]–[28], [32], [33].

In the present study, we report the first application of ATMT for insertional mutagenesis of *V. dahliae*. We established a laboratory unimpaired root dip-inoculation method to detect insertional mutants with altered pathogenicity in cotton. Sequence analysis of the tagged genes led to the isolation of five different putative pathogenicity genes. The tagged gene in one pathogenicity mutant, with a single-copy insertion and greatly delayed formation of melanized microsclerotia on PDA agar medium, is a glutamic acid-rich protein 1 (*VdGARP1*); it shared no significant similarity to any known annotated gene. Over-expression of the *VdGARP1* genomic DNA sequence restored the *vdgarp1* mutant growth morphology and pathogenicity. The expression of *VdGARP1* in wild type V529 strain was induced or inhibited by different stress agencies and conditions, and it was induced by cotton root extract in liquid culture medium. Overall, our findings indicate that insertional mutagenesis by ATMT, together with our unimpaired root dip-inoculation approach, is a valuable tool for the genome-wide analysis of gene function and identification of pathogenicity genes in this important cotton pathogen.

## Results

### ATMT mutant morphologies and insertion identification

**Figure 1:**
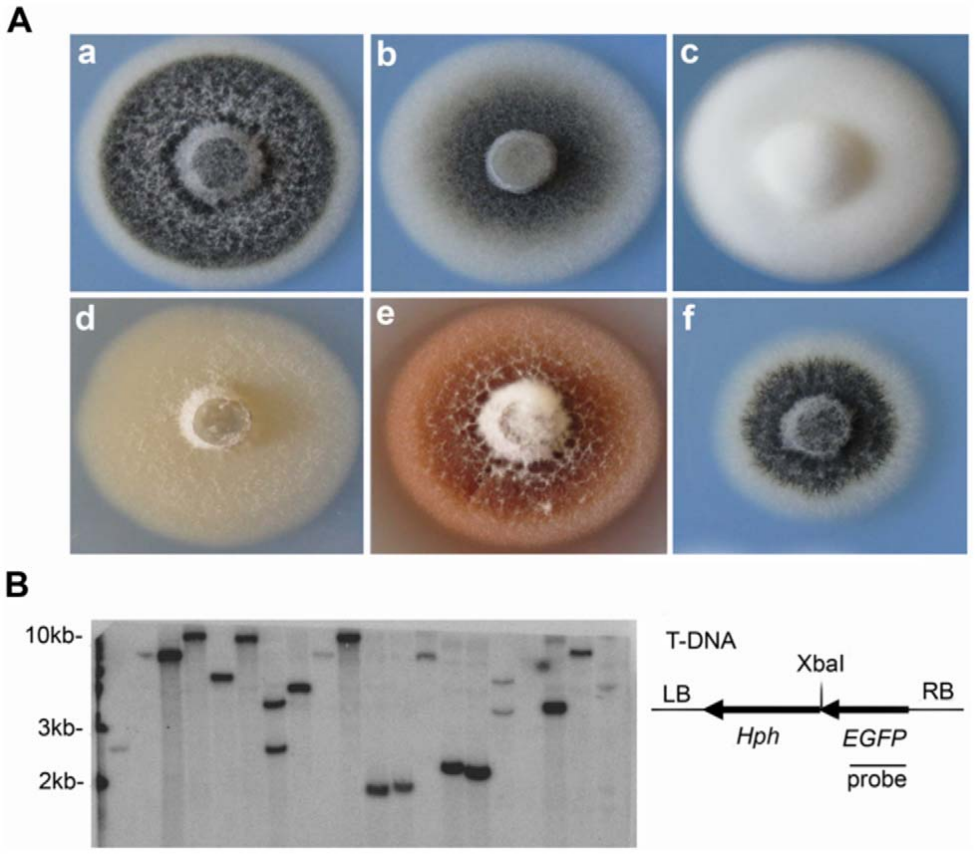
T-DNA insertion mutant morphologies of V592 and insertion identification. (A) Colony morphologies of wild type V592 (a) and T-DNA insertion mutants (b to f), classified as intermediate type (b), hyphal type (c) and velum type (d), as well as special types such as red pigment (e) and slow-growing rate (f) mutants. (B) Identification of T-DNA insertional copy numbers. Genomic DNA isolated from one hundred randomly selected transformants was digested with *Xba*I for DNA gel blot analysis. Representative result with twenty mutants is shown. Hybridization was performed with ^32^P-labeled EGFP-specific DNA probe as shown at right.

Strain V592 of *V. dahliae*, isolated from cotton in Xinjiang, China, was used to create T-DNA insertional mutants yielding 2,323 primary hygromycin-resistant mutants. Changes in growth rate and pigmentation traits of these mutants were observed directly on Potato Dextrose Agar (PDA) medium. Most mutant colonies (92.80%) showed no distinct differences in growth and pigmentation traits when compared to wild type V592 (Figure 1A, a). Some mutants (4.18%) showed normal mycelial growth but reduced microsclerotial development, described as the intermediate type (Figure 1A, b). Some mutant (2.67%) colonies exhibited a total loss of melanized development but had rapid growth and a denser appearance than that of V592, as hyphal type (Figure 1A, c). Other mutants (2.2%) displayed “unwettable” dipcoat-like phenotype, as velum type (Figure 1A, d). One mutant showed diffuse red pigment (Figure 1A, e), and three displayed slow development of mycelia but normal formation of microsclerotia (Figure 1A, f). The transformants appeared to be mitotically stable because mono-conidial cultures from one hundred randomly selected transformants retained hygromycin resistance and mutant phenotypes after being subcultured three times on PDA medium. Over 85% of the 100 transformants were estimated, by DNA blot analysis, to have a single copy of the T-DNA integrated into the genome (Figure 1B). The multiple and various degrees of mutant traits indicated that mutant phenotypes resulted from *V*. *dahliae* genes affected by T-DNA random insertion.

### Pathogenicity mutant screen

**Figure 2:**
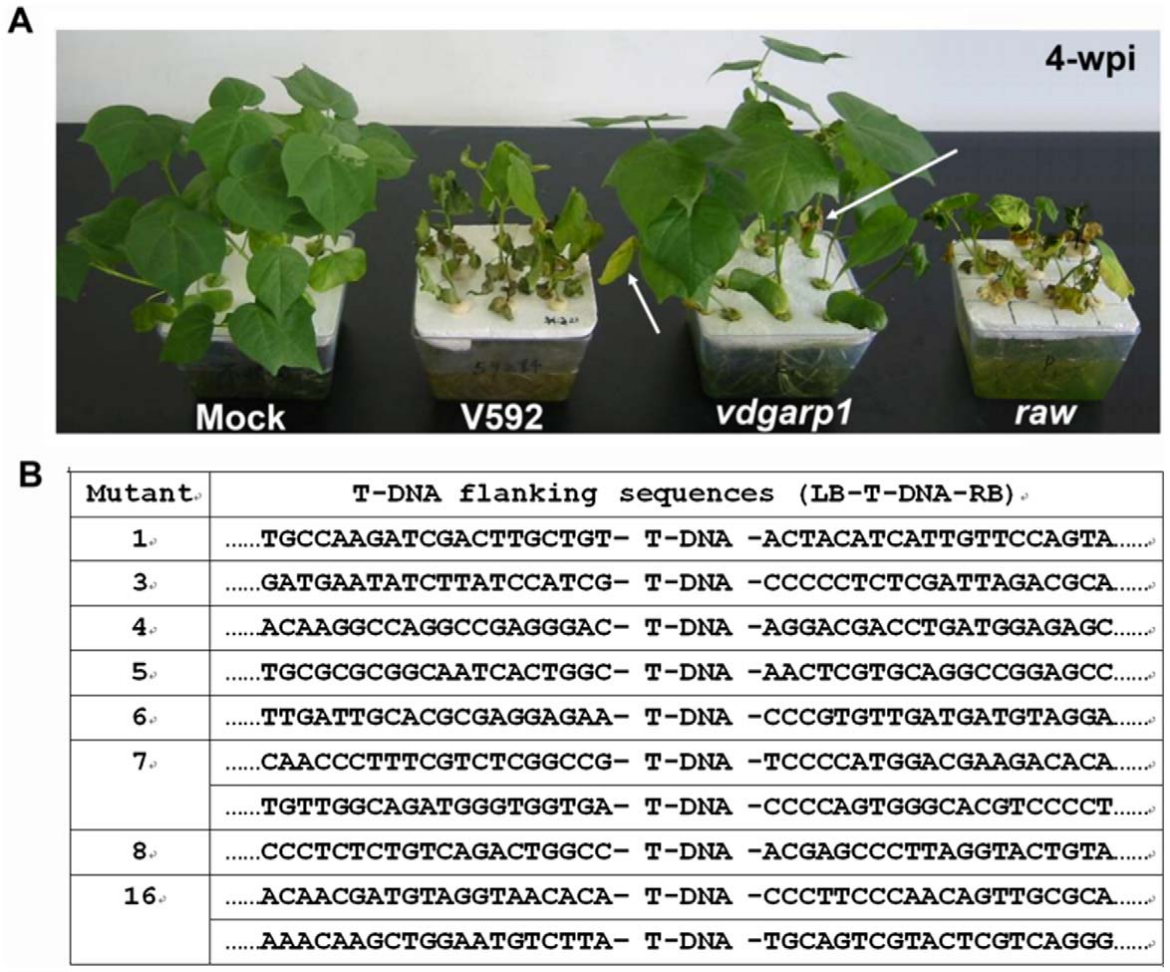
Disease symptoms and T-DNA border sequences of pathogenicity mutants. (A) Disease symptoms of wild type V592 and pathogenicity mutants, *vdgarp1* and *raw*, on cotton plants. Arrows indicated a reduced degree of wilting leaf in some old leaves of *vdgarp1-*infected seedlings. Photographs were taken at four weeks post-inoculation. (B) Different sequences flanking T-DNA border sequences in pathogenicity mutants.

Among the multiple mutant phenotypes, pathogenicity-defective mutants were our primary focus in this study. The regular procedure for infection of plants with the soil-borne pathogen is to uproot soil-grown plants, incubate the roots in a conidial suspension, and then replant the plants in fresh soil. To avoid damaging the roots and better to mimic natural infection conditions, we first developed a laboratory unimpaired root dip-inoculation method to assess the insertional mutant pathogenicities in cotton (see Materials and Methods). Twelve, two-week-old cotton seedlings were root dip-inoculated with spores from wild type V592. Leaf wilt was first visually apparent on leaves at two weeks post-inoculation (wpi), and the whole leaf was dried out, epinastic and scorched by 3 wpi. At this time, cotyledons were lost and roots became brown and slimy. By 4 wpi, whole seedlings dried out and completely collapsed (Figure 2A). Disease symptoms did not occur on cotton seedlings with mock inoculation during the whole two-month experiment period (Figure 2A). Our results suggested that the unimpaired root dip-inoculation appeared accurately to reflect the disease progress of a natural infection of cotton by *V. dahliae*.

Next, we examined the effects of about 500 mutations on pathogenicity. For each mutation, twelve cotton seedlings were inoculated. At approximately 4 wpi, symptoms produced by most (98%) insertional mutants were indistinguishable from those caused by V592. Five mutants caused delay and reduced induction of symptoms, with reduced rates of wilting and loss of cotyledons. Some late-growth (upper layer) leaves of infected seedlings maintained normal developmental phenotypes. Two mutants showed great reduction of infection (Figure 2A, *vdgarp1*, see section below); no disease symptoms developed during the early growth stages of cotton seedlings inoculated with these mutants. By 4 wpi, a reduced degree of leaf wilting was observed in some old leaves of these seedlings (Figure 2A, arrow), which grew vigorously with biomasses and heights similar to those of seedlings with mock inoculation. One mutant infection caused rapid leaf wilt (named as *raw*) and complete leaf drying at 2 wpi, almost two weeks earlier than observed in wild type V592 infection (Figure 2A). These eight mutant colonies were subjected to thermal asymmetric interlaced (TAIL) PCR [34] and sequencing. We identified different sequences flanking the T-DNA border sequences in these mutants (Figure 2B), consistent with their T-DNA insertion DNA blot analysis (Figure 1B), indicating that change in infectivity of the mutants was the result of insertional knockout rather than the integrated T-DNA itself. Detailed analysis of one of the mutants in gene cloning and functional complementarity is described below.

### Disruption of a glutamic acid-rich protein gene causes significantly delayed development of melanized microsclerotiain of *V. dahliae*

**Figure 3:**
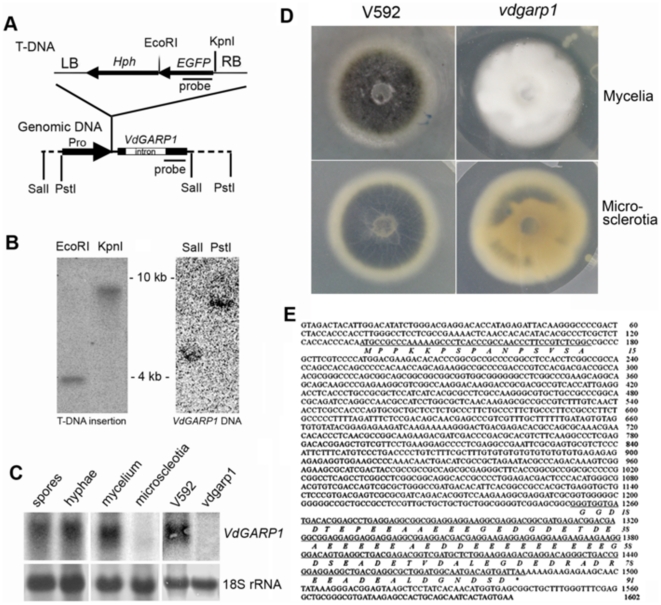
Characterization of *VdGARP1* gene. (A) Schematic diagrams of T-DNA and *VdGARP1* genomic DNA construct. T-DNA insertional position upstream 358 bp from initiation codon in *VdGARP1* genomic was shown. (B) DNA gel blot analysis of T-DNA insertional copy number from *vdgarp1* mutant (left panel), and *VdGARP1* DNA copy number from V592 (left panel) with ^32^P-labeled T-DNA-specific or *VdGARP1*-specific DNA probe shown in (A). (C) Identification of expression of *VdGARP1* mRNA in spores, hyphae, mycelia and microscleotia of V592, as well as in mycelia of V592 and *vdgarp1* mutant, with ^32^P-labeled *VdGARP1*-specific DNA probe shown in (A). rRNAs stained with methylene blue trihydrate were used as a loading control. (D) Morphology of V592 and *vdgarp1* colony. *vdgarp1* mutant displayed denser appearance mycelia with great delayed and reduced microsclerotial development on PDA agar medium compared to that of V592. (E) Full length *VdGARP1* cDNA sequence obtained by 5′-rapid amplification of cDNA end (5′RACE) and 3′RACE, revealing *VdGARP1* gene encodes an glutamic acid-rich protein from a gene with a 1076 bp intron sequence.

The two mutants that displayed great reduction of infectivity (Figure 2A) were verified as pathogenicity mutants after two rounds of testing in a secondary screen of cotton seedlings. One of these was confirmed to have a single copy of the T-DNA (Figure 3B) integrated in the promoter of a hypothetical protein gene (Figure 3A), by comparison to sequences of VdLs.17, a *V. dahliae* isolate from lettuce (http://www.broadinstitute.org/annotation/genome/verticillium_dahliae/Blast.html). The growth rate of the mutant colony showed no significant difference from that of V592; however, the mutant colony exhibited a denser appearance with greatly delayed and reduced microsclerotial development on PDA agar medium (Figure 3D).

To confirm further the transcript RNA that encoded the predicted protein in V592, 5′RACE and 3′RACE were performed. These revealed a 526-bp cDNA sequence, with 131 bp upstream of the predicted translation start site and a polyadenylation site 119 bp downstream of the translation stop codon, encoding a polypeptide of 91 amino acids (enriched with 31% glutamic acid) that was predicted (Figure 3D), using the signal peptides prediction program [35], to be non-secretory. Neither nucleotide nor amino acid sequences shared any similarity to any annotated gene in GenBank. This glutamic acid-rich sequence that contains a 1076-bp intron sequence in V592 was confirmed to be single-copy by DNA blot analysis and PCR sequencing (Figure 3B and 3E).

The expression of the hypothetical protein-coding mRNA in V592 was verified by RNA blot analysis with total RNA extracted from hyphae and spores collected from liquid Czapek-Dox medium, as well as from mycelia collected from PDA agar medium, but not from microsclerotia collected from the infertile nutrient agar medium [18] (Figure 3C). These results indicate that expression of the transcript is organ-specific. There was no corresponding RNA signal detected in the mutant hyphae sample, confirming that it is a null mutant (Figure 3C). The hypothetical protein contained thirty-one glutamic acid residues; therefore, it was named glutamic acid-rich protein 1 (*VdGARP1*), with the mutant named *vdgarp1*.

### Ectopic over-expression of *VdGARP1* rescues melanized microsclerotial formation and pathogenicity of the *vdgarp1* mutant

**Figure 4:**
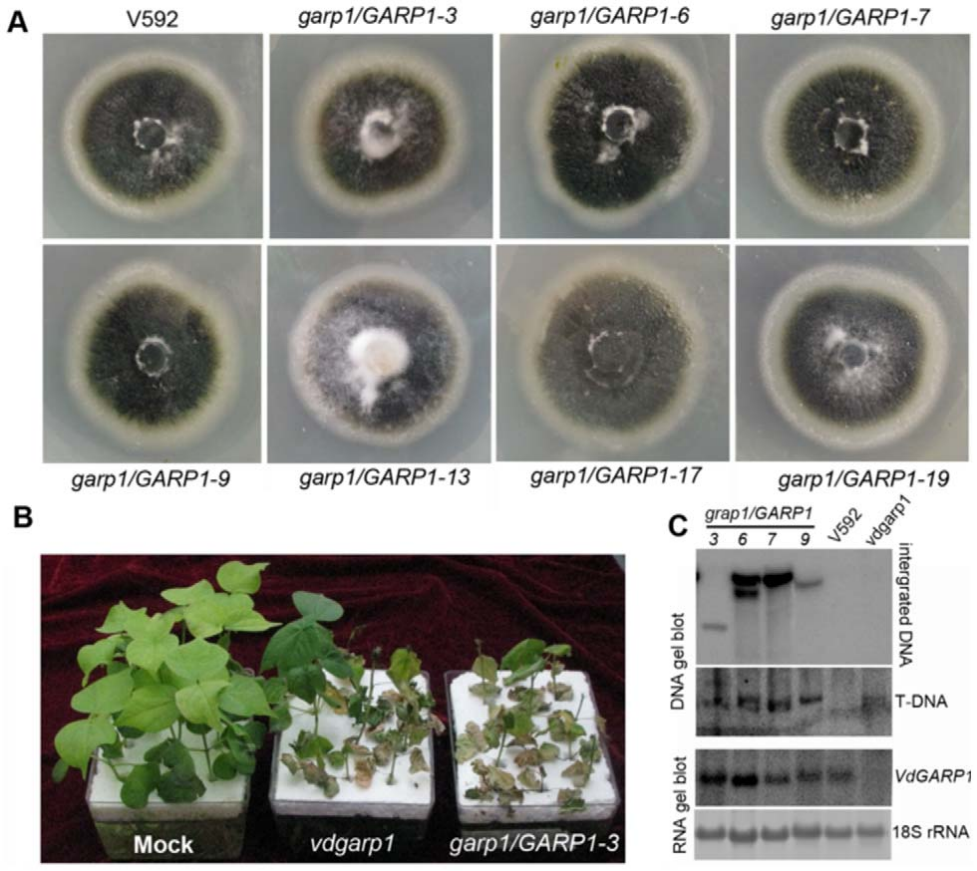
Analysis of ectopic over-expression of *VdGARP1* in *vdgarp1* mutant. (A) Seven individual hygromycin-resistant transformants (*garp1/GARP1*) shown rescued melanized microsclerotial formation similar to that of V592. (B) *garp1/GARP1* recovered infectivity comparable to that of V592. Representative of *garp1/GARP1-3* was shown. (C) DNA and RNA gel blots analysis confirmed that the transformants containing different ectopic insertions of the *VdGARP-* complementing construct and the original T-DNA insertion *vdgarp1* background, as well as ectopic over-expression of *VdGARP1* RNA. DNA and RNA extracted from V592 and *vdgarp1*, respectively, were used as controls, rRNAs stained with methylene blue trihydrate were used as a loading control.

To examine the severe inhibition of melanized microsclerotial development and defective pathogenicity that resulted from the loss of *VdGARP1* function, we introduced a copy of the *VdGARP1* genomic sequence of wild type V592, including the upstream 1600-bp putative promoter region, into the mutant *vdgarp1* by ATMT. Twenty individual hygromycin-resistant transformants, purified by single-spore isolation, were obtained. Among them, seven colonies displayed melanized microsclerotial development comparable to that of V592 (Figure 4A). This frequency (35%) of ectopic integration suggests operability in complementation of this mutant phenotype. Lower frequency (4.3%) has been reported for the *VDH1* gene in ectopic integration into the *vdh1* mutant in *V. dahliae* [23]. Four complementary colonies (*garp1*/*GARP1*) were assayed for infectivity. All of them recovered infectivity comparable to that of wild type V592 (Figure 4B). DNA gel blot analysis confirmed that the transformants contained ectopic insertions of the *VdGARP1-*complementing construct and the original T-DNA insertion of the *vdgarp1* background (Figure 4C). RNA gel blot analysis also confirmed expression of *VdGRP1* RNA (Figure 4C). There is no gene to the 5′ side of the *VdGRP1* gene confirmed in wild type V592 by RNA gel blot analysis using the promoter sequence of the *VdGRP1* gene as probe (data not shown). Taken together, these results indicate that specific knock-out the *VdGARP1* gene indeed results in severe inhibition of melanized microsclerotial development and lost infectivity of V592.

### Analysis of expression of *VdGARP1* transcript under stress condition

**Figure 5:**
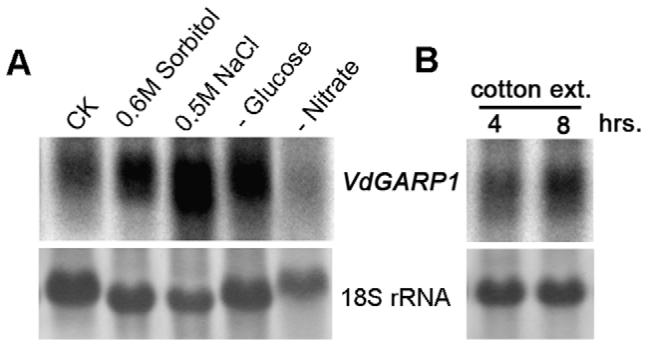
RNA gel blot analysis of expression of *VdGARP1* transcript under different conditions. (A) Expression of *VdGARP1* transcript under stress conditions by adding sorbitol or NaCl to three-day-old V592 liquid culture, or by transfer three-day-old V592 liquid culture to liquid medium lacking glucose or nitrate. (B) Expression of *VdGARP1* mRNA stimulated by cotton, by adding cotton root extract to three-day-old V592 liquid culture. Co-culture times are as indicated. rRNAs stained with methylene blue trihydrate were used as a loading control. Hybridization was performed with ^32^P-labeled *VdGARP1*-specific DNA probe as shown in Figure 3A.

One striking phenotype of the *vdgrp1* mutant was its enhanced hyphal development (Figure 3D). This suggested that knocking out the *VdGARP1* gene resulted in reducing sensitivity of V592 to normal growth conditions to develop microsclerotia. We then directed our efforts to determine whether stress could stimulate expression of *VdGARP1.* For this purpose, a patch of colony on agar medium was picked to culture in liquid medium for three days; different stress conditions were then applied by adding sorbitol or NaCl, or by transfer to liquid medium lacking glucose or nitrate, and the cells were cultured for an additional eight hours. Figure 5A shows that accumulation of *VdGARP1* mRNA increased by incubation with sorbitol and NaCl, as well as in a medium lacking glucose (Figure 5A). However, accumulation of *VdGARP1* mRNA decreased in a medium lacking nitrate (Figure 5A). These results suggested that drought and salt as well as a lack of saccharides stimulated expression of *VdGARP1* mRNA, while a lack of nitrate inhibited expression of *VdGRP1* mRNA.

To examine whether induction of *VdGRP1* mRNA was also stimulated by cotton, cotton root extract was added to three-day-old V592 liquid culture, which then continued to culture for four to eight hours. Northern blot analysis showed that *VdGARP1* mRNA accumulation increased with time of co-culture with cotton root extract (Figure 5B). This suggests that the *VdGARP1* gene may play a role in inducing infection and promoting microsclerotial development in infected cotton roots, wherein some host component(s) might stimulate *VdGARP1* expression.

### Correlation between expression of *VdGARP1* mRNA and formation of melanized microsclerotia under near-UV light

**Figure 6:**
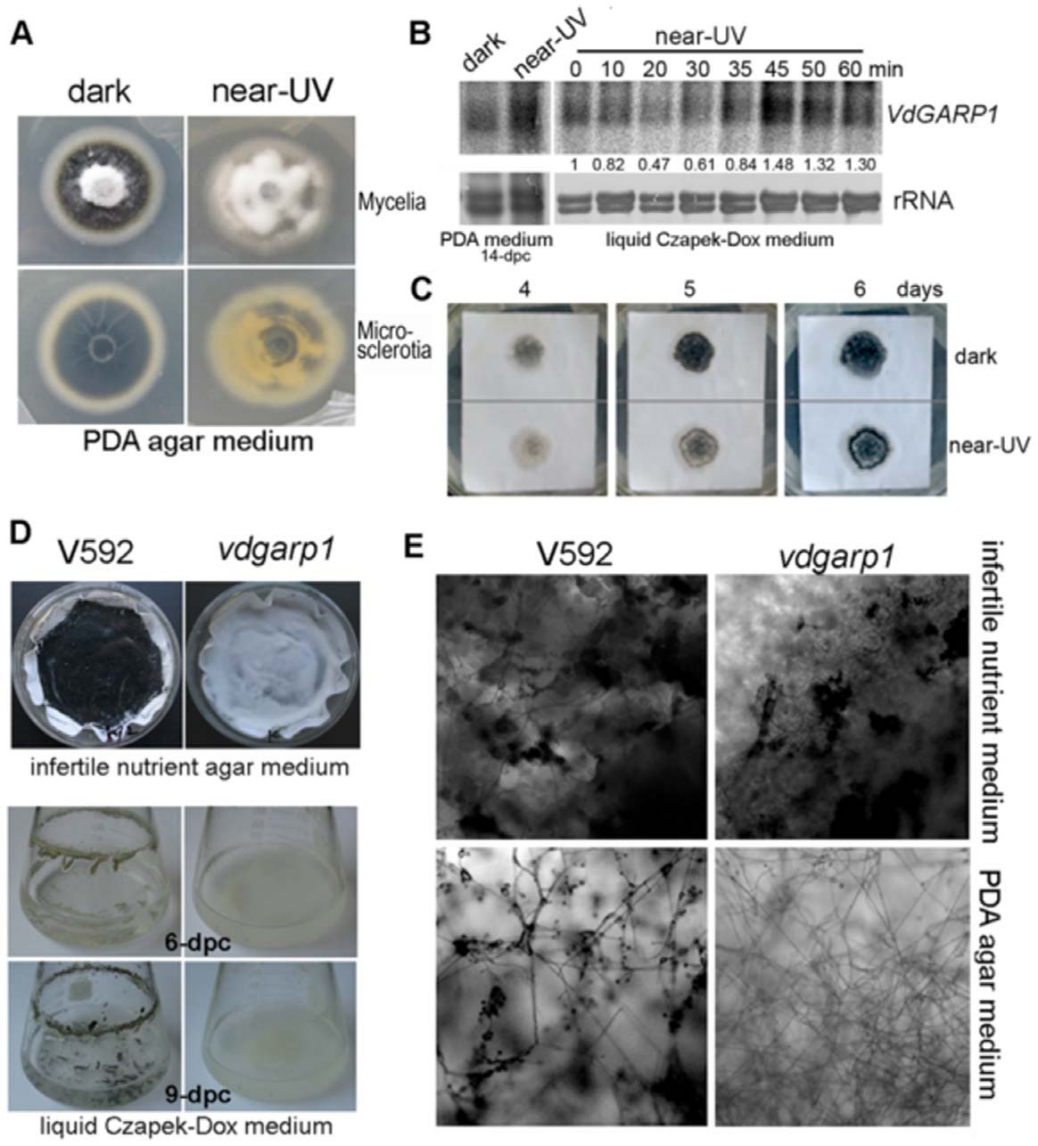
Analysis of expression of *VdGARP1* mRNA and formation of melanized microsclerotia under near-UV light. (A) Mycelial growth and melanized microsclerotial production under normal dark-grown conditions and near-UV light, photos were taken at 14 days post-culture on PDA agar medium. (B) RNA gel blot analysis of *VdGARP1* mRNA expression of V592 on PDA agar medium (left panels) and time course from liquid culture (right panels) exposure to near-UV light. rRNAs stained with methylene blue trihydrate were used as a loading control. Quantification of VdGARP1 mRNA relative to total RNA is shown below of the panel. The value of 0 hour treatment was arbitrarily designated as 1. (C) Development of melanized microsclerotia of V592 in infertile nutrient conditions. Photographs were taken at 4-, 5- and 6-dpc. (D, E) Melanized microsclerotial production of V592 and the *vdgarp1* mutant spores were grown in either liquid Czapek-Dox medium or infertile nutrient agar medium. Melanized microsclerotia under 400x microscope (E).

Near-UV light has been reported to inhibit microsclerotial formation [23]. We therefore assessed the effects of near-UV light on microsclerotial development and *VdGARP1* mRNA expression. Increased accumulation of *VdGARP1* mRNA was detected when V592 colonies on PDA agar medium were exposed to near-UV light for 14 days (Figure 6B). However, vigorous mycelial grown with some patches of melanized microsclerotial production was observed compared to that from normal dark-growth conditions (Figure 6A). To enable more precise detection of effects of near-UV light on expression of *VdGARP1* mRNA, a three-day-old liquid culture containing hyphae and spores was poured into plates and kept under continuous exposure to near-UV light. Time course RNA gel blot analysis showed that accumulation of *VdGARP1* mRNA decreased in the first 30 minutes and began to increase under near-UV light (Figure 6B), suggesting dynamic regulation of *VdGARP1* mRNA expression by near-UV light. Reduced expression of *VdGARP1* mRNA in response to near-UV light at early time points may be partially responsible for the vigorous mycelial growth on agar media (Figure 6A). To investigate the effect of near-UV light on development of melanized microsclerotia in infertile nutrient conditions, confluent spores of V592 were grown on cellulose membranes overlaid on basal agar medium supplemented with 20 mM sodium nitrate [18], a condition that simulated infertile nutrients and induced near-synchronous development of melanized microsclerotia. Near-synchronous development of melanized microsclerotia was observed at 4 days post culture (dpc) in dark-grown conditions but not in cultures exposed to near-UV light, in which more mycelia growth was observed (Figure 6C). At 5 to 6 dpc, melanized microsclerotial production was also clearly observed in cultures exposed to near-UV light, with less extension than that in dark-grown culture (Figure 6C). These results indicated that near-UV light slightly delayed but did not inhibit melanized microsclerotial development of V592 under infertile nutrient conditions.

Finally, we examined the melanized microsclerotial production of the *vdgarp1* mutant under infertile nutrient conditions. The same amounts of V592 and *vdgarp1* mutant spores were grown in either infertile nutrient agar medium or liquid Czapek-Dox medium. Melanized microsclerotial formation was observed for both wild type V592 and *vdgarp1* mutant spores in infertile nutrient agar medium, with vigorous mycelial growth in the *vdgarp1* mutant but not in V592 (Figure 6D and 6E). However, melanized microsclerotial production was observed for V592 but not for *vdgarp1* mutant in liquid Czapek-Dox medium at 6 to 9 dpc (Figure 6D and 6E). These results suggested that *VdGARP1* transcript was required for sensing circumstances, such as nutrient-limitation, to develop melanized microsclerotia. However, under extreme infertile nutrient conditions, *VdGARP1* was not necessary for melanized microsclerotial formation and development; this was consistent with the fact that no *VdGARP1* mRNA was detected in melanized microsclerotia (Figure 3C).

## Discussion

In this report, we constructed a T-DNA insertional mutagenesis library of a virulent defoliating isolate of *V. dahliae*, from cotton that originated in Xinjiang, China. Different morphologies in growth rates and pigmentation traits of mutants were obtained (Figure 1A). The transformants were mitotically stable, and most transformants were estimated to have a single copy of the T-DNA integrated into the genome (Figure 1B). This result was desirable because multiple T-DNA insertions could not be separated through genetic segregation because *V. dahliae* lacks a sexual stage. Single site insertion in the genome also facilitated the recovery of tagged genes from such fungi [36]. Our results suggested that ATMT could be further used to create a large-scale *V. dahliae* library of insertional mutants for genome-wide functional analysis, as was done for *M. oryzae* [28], though manipulation of infection with this soil-borne *V. dahliae* would be much more difficult.

The infection assay for *M. oryzae* and other foliar pathogens is normally carried out using conidial suspensions to drop onto cut leaves or spray directly onto plants [37]. For infection with the soil-borne pathogen, plants normally need to be up-rooted from the soil and replanted into fresh soil after co-incubation of roots with conidial suspensions. This process can hardly avoided damage to roots and could not accurately reflect real functions of the genes of the pathogen of interest, particularly their pathogenicity. In this study, we established a laboratory unimpaired root dip-inoculation method for inoculation and found that it accurately reflected the progress of a natural infection of cotton by *V. dahliae* V592 (Figure 2A). This greatly facilitates the manipulation of infection with soil-borne *V. dahliae*.

Using the T-DNA insertional mutagenesis library of V592 and the unimpaired root dip-inoculation method, we detected several insertional mutants with altered pathogenicity in cotton. One glutamic acid-rich protein 1 (*VdGARP1*), which shared no significant similarity to any known annotated gene. It plays a role in pathogenicity, presumably stimulated by cotton root component(s) in response to changes in infection environment, to induce formation of melanized microsclerotia. The fact that expression of *VdGARP1* in V529 was induced by drought and salt stress as well as in low-carbohydrate conditions (Figure 5A) suggests that *VdGARP1* mRNA plays an important role in stress responses in the infected cotton roots under certain conditions to promote melanized microsclerotial formation. The formation of microsclerotia is beneficial in the life cycle of the fungus by transferring from a saprophytic to dormant state for long term survival. At later time points in infection, development of melanized microsclerotia in extreme infertile nutrient conditions in infected roots was no longer required as was found in infertile nutrient conditions (Figure 6D). We could not rule out the possibility that cotton root component(s) might reduce expression of *VdGARP1* mRNA early in infection to favor vigorous hyphal growth, as was observed with near-UV light treatment (Figure 6B). This would accelerate hyphal penetration of host roots in the initiation of the parasitic stage of the life cycle of *V. dahliae*.

From *V. dahliae* tomato isolate, two genes, *VMK1* and *VDH1*, involved in microsclerotial development have been identified. *VMK1* encodes a mitogen-activated protein (MAP) kinase that is essential for pathogenicity, suggesting that the MAP kinase-mediated signaling pathway has a conserved role in fungal pathogenicity [21]. *VDH1* is not required for pathogenicity [22], [23]. *VDH1* is a hydrophobin gene that contains eight cysteine residues with a conserved spatial distribution [22]; nutrient starvation, such as a lack of nitrate or glucose, increases its expression, while near-UV light shows no effect on its expression [23]. Unlike *VDH1*, *VdGARP1* accumulation is greatly induced in media lacking glucose but inhibited in media lacking nitrate (Figure 5A). Near-UV light also shows dynamic regulation of *VdGARP1* expression. Hydropathicity analysis (http://www.expasy.org/tools/protscale.html) [38] suggests VdGARP1 to be a hydrophilic protein. A significant surplus of negative charges is localized in this protein, which contains 31 glutamate and 17 aspartate residues (Figure 3E). The protein contains only two arginine and two lysine residues, which are far from being able to compensate for the negative charge. Together with hydropathicity analysis, we reason that VdGARP1 belongs to the class of intrinsically unstructured proteins. Such proteins, for example glutamic acid-rich proteins (GARPs) in vertebrate rod photoreceptors, were suggested to be loose coils with low-affinity but high-capacity Ca^2+^ binding [39]. Further investigation is necessary to determine whether the high-charge-density VdGARP1 protein also possesses Ca^2+^ binding capacity and the effect of this capacity on response to nutrient attenuation in infected plant cells to promote melanized microsclerotial formation.

## Materials and Methods

### Fungal isolates and culture conditions

A virulent defoliating *V.dahliae* isolate V592 from cotton originated in Xinjiang, China, was used in this study. This isolate and its transformants were stored at -80°Cin the form of microconidial suspension in 20% glycerol. Cultures were reactivated on Potato Dextrose Agar (PDA) medium (Becton, Dickinson and Company). Microsclerotial formation assays were performed on infertile nutrient medium, that overlayed a cellulose membrane on basal agar medium supplemented with 20 mM sodium nitrate to create a condition simulated near-synchronous development of melanized microsclerotia [18]. Conidia production for infection assays and germination tests were cultured in liquid Czapek-Dox medium (30 g/L Sucrose, 3 g/L NaNO_3_, 0.5 g/L MgSO_4_-7H_2_O, 0.5 g/L KCl, 100 mg/L FeSO_4_-7H_2_O, 1 g/L K_2_HPO_4_, pH 7.2).

### DNA cloning and sequencing

A thermal asymmetric interlaced PCR (TAIL-PCR) protocol [40] was used for cloning genomic DNA flanking inserted T-DNA from the insertion mutants. The right border primers (RB-1, -2, and -3) and the left border primers (LB-1, -2, and -3) was used as previously described [40]. Eleven arbitrary degenerate primers were designed such that their melting temperatures (T_m_'s) would ensure maximum thermal asymmetric priming and ensure cloning and sequencing of genomic DNA flanking T-DNA:

AD1: (AGCT)TCGA(GC)T(AT)T(GC)G(AT)GTT;

AD2: (AGCT)GTCGA(GC)(AT)GA(AGCT)A(AT)GAA;

AD3: (AT)GTG(AGCT)AG(AT)A(AGCT)CA(AGCT)AGA;

AD4: TG(AT)G(AGCT)AG(AT)A(AGCT)CA(GC)AGA;

AD5: AG(AT)G(AGCT)AG(AT)A(AGCT)CA(AT)CA(AT)AGG;

AD6: CA(AT)CGIC(AGCT)GAIA(G/C)GAA;

AD7: TC(GC)TICG(AGCT)ACIT(AT)GGA;

AD8: GC)TTG(AGCT)TA(GC)T(AGCT)CT(AGCT)TGC(;

AD9: (AT)CAG(AGCT)TG(AT)T(AGCT)GT(AGCT)CTG;

AD10: TCTTICG(AGCT)ACIT(AGCT)GGA;

AD11: TTGIAG(AGCT)ACIA(AGCT)AGG;

A thermal cycler (MJ Research/Bio-Rad) was used for the TAIL-PCR. The tertiary TAIL-PCR product of each transformant showing the highest intensity was purified using QIAquick columns (Qiagen) and sequenced (Invitrogen). The innermost specific primer, either RB-3 or LB-3, was used as the sequencing primer. By comparing sequences of VdLs.17, a *V. dahliae* isolate from lettuce (http://www.broadinstitute.org/annotation/genome/verticillium_dahliae/Blast.html), the T-DNA insertion site was detected.

For obtaining the full-length cDNA sequence, 5′ RACE and 3′ RACE was performed according to the manufacturer's instructions (Ambion, First Choice RLM-RACE Kit). The 5′ RACE gene specific primer (5GSPout:5′-TCATCGCCGTCCTCGCCTTC-3′ and 5GSPinner:5′-CTCCTCTTCCTCGTCGTCCT-3′) and the 3′ RACE gene specific primer (3GSPout:5′-TGATGACACGGAGCCTGAG-3′ and 3GSPinner:5′-AAGGCGAGGACGGCGATGA-3′) were designed based on the predicted sequence.

To generate the *VdGARP1* complementation construct, a 3.7-kb *VdGARP1* genomic DNA sequence containing the predicted promoter was amplified from V592 DNA, with primers VdGARP1DNA-s (5′-GTCTAGATATGCCTTGATGACGAGGTT-3′, *Xba*I site is underlined) and VdGARP1DNA-a (5′-CGGATCCCTGCTTGTTCGGTTCTTCGTTT-3′, *Bam*HI site is underlined), and then ligated into the pGEM-T easy vector(Tiangen) to generate pGEM-VdGARP1. *Bam*HI-*Xba*I fragment of pGEM-VdGARP1 was then cloned into pSULPH-EGFP [41] digested with *Bam*HI and *Xba*I to give pSULPH-VdGARP1 for complementary transformation.

### Nucleic Acid extraction and blotting

Fungal isolates were grown in the liquid Czapek-Dox medium for 3–5 days with shaking at 200 rpm, 28°C, dark condition, and the resulting mycelium was harvested by centrifugation at 12000 rpm for 1 min. The collected mycelium was stored at −80°C until DNA and RNA was extracted.

For genomic DNA isolation, a CTAB protocol was used as previously described [42]. Twenty µg of genomic DNA was completely digested and separated by electrophoresis on an agarose gel and transferred onto a nitrocellulose membrane. DNA gel blots were performed according to as previously described [43] with specific probes labeled with ^32^P using the Random Prime Labeling System Redi Prime™II (GE Healthcare, Piscataway, NJ, USA). The EGFP probe (for detection of T-DNA insertion) was amplified from the vector pSULPH-GFP [41], with primers EGFP-s (5′-ATGGTGAGCAAGGGCGAGGAG-3′) and EGFP-a (5′-TTACTTGTACAGCTCGTCCATGCCG-3′). The ToxA probe (for detection of complementing DNA) was amplified from the vector pSULPH-GFP, with primers ToxA-s (5′- CTATATTCATTCATTGTCAGCTATC-3′) and ToxA-a (5′-GATTGGAATGCATGGAGGAGTTC-3′).

Total RNA was isolated as previously described [43]. Fifteen µg total RNA was used for RNA gel blot analysis. Hybridization was carried out as described [43] with *VdGARP1*-specific DNA probe, amplified by reverse transcription-PCR (RT-PCR). The first strand of cDNA was synthesized with 1 µg of total RNA in a 20-µl RNA PCR mixture (TaKaRa). After a 10-fold dilution, 1 µl of the RT products was used as a template in PCR amplification with primers VdGARP1-s (5′-ATGCCGCCCAAAAAGCCCTCACCCG-3′) and VdGARP1-a (5′-TTAATCACTGTCATTGCCATCCAGC-3′).

### *Agrobacterium tumefaciens*-mediated transformation (ATMT)

ATMT was used as previously described [40] with some modifications. *A. tumefaciens* strain EHA105, containing an appropriate binary vector (PLL16, constructed by cloning a *Sac*I-*Kpn*I fragment from pCX12 [44] containing the *EGFP-TtrpC* construct into pBHT2 [40]; pSULPH-VdGARP1), was grown at 28°C for 2 days in LB Medium (Luria-Bertani Medium) supplemented with kanamycin (50 µg/ml). The *A. tumefaciens* cells were diluted to (optical density) OD600  = 0.2 in induction medium (IM) [40], in the presence of 200 µM acetosyringone (AS) (BioDee). The cells were grown for an additional 6 hours before mixing them with an equal volume of a conidial suspension of V592 (1×10^7^ conidia per ml). The mix (200 µl per plate) was plated on a 0.45-µm pore, 45-mm diameter nitrocellulose filter (Whatman) and placed on cocultivation medium (same as IM except that it contains 5 mM glucose instead of 10 mM glucose) in the presence of 200 µM AS and 40 mM MES (pH 5.3) (BioDee). After co-incubation at 26°C for 36–48 hours, the cultures were washed with 2 ml sterile water per plate, and then transferred to Defined complex medium (1.70 g/L Yeast Nitrogen Base without amino acids, 2 g/L Asparagine, 1 g/L NH_4_NO_3_, 10 g/L Glucose, pH to 6.0 with Na_2_HPO_4_) containing hygromycin B (for PLL16, 75 µg/ml) (Roche) or Chlorimuron-ethy1 (for pSULPH-VdGARP1, 100 µg/ml) (Wuhan Xinhuayuan, China) as a selection agent for transformants and cefotaxime (200 µg/ml) (BioDee) and carbenicillin (200 µg/ml) (BioDee) to kill the Agrobacterium tumefaciens cells. Individual transformants were transferred into PDA medium containing hygromycin B or Chlorimuron-ethy1and incubated until conidiogenesis. Conidia of individual transformants were suspended with sterile water and plated on PDA medium. Spores from these monoconidial cultures were stored in 20% glycerol at −80°C until further analysis.

### Infection assays

Cotton plants (cv. Xinluzao NO. 16) were used in infection assays to evaluate the effect of *V.dahliae* isolate V592 mutation on virulence. Mutants generated from V592 were evaluated for their virulence on cotton (cv. Xinluzao NO. 16), using our “laboratory unimpaired root dip-inoculation method”. To prepare inocula, fungal cultures grown for 5 days in the liquid Czapek-Dox medium were passed through several layers of cheesecloth (to remove mycelia), and the conidial concentration was adjusted to approximately 1×10^7^ conidia per ml. 12 Seedlings per pot were planted in MS liquid medium (Murashige and Skoog medium) in an environmentally controlled growth room at 26±1°C, 60–70% relative humidity, on a 16-h light/8-h dark cycle, for about two weeks. At the third true leaf stage, the seedlings were then inoculated by immersing their roots into the conidial suspension for 50 minute. The seedlings were then put back to the 1/10 MS liquid medium. Disease progress was recorded over time for two months of experiment period. Disease severity was counted by the percent of leaves that showed wilting symptom at each time point. Infection assay for each mutant colony was repeated at least three times.
